# Supplementary figures and images for: Information acquisition and cognitive processes during strategic decision-making: Combining a policy-capturing study with eye-tracking data
Source: PLoS One. 2022 Dec 1;17(12):e0278409. doi: 10.1371/journal.pone.0278409 (PMC9714927; doi:10.1371/journal.pone.0278409)

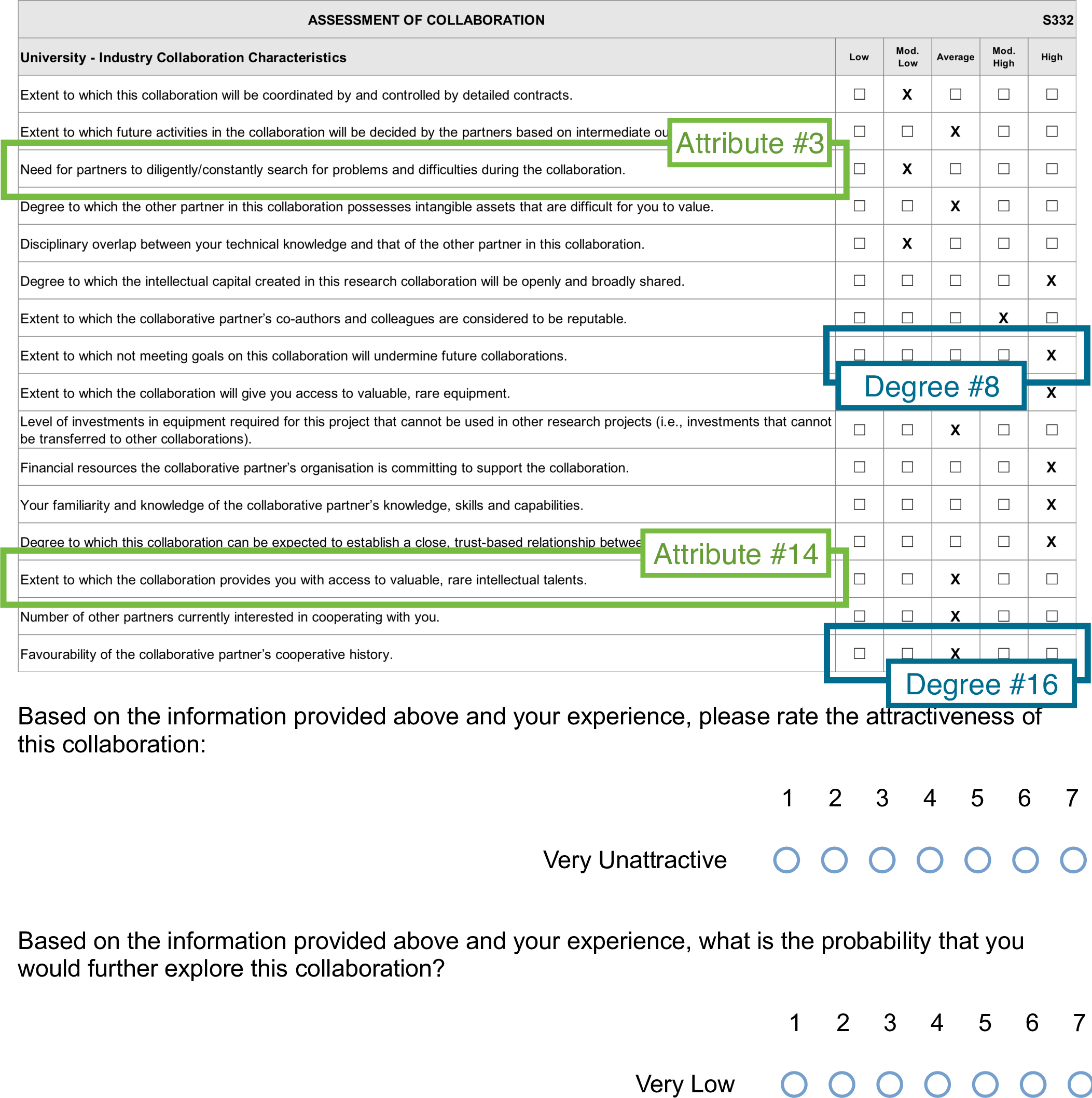

Supplement: S1 Fig — An example of the structure of one of the 30 scenarios and the Areas of Interest distribution. (TIF) [file pone.0278409.s001.tif]

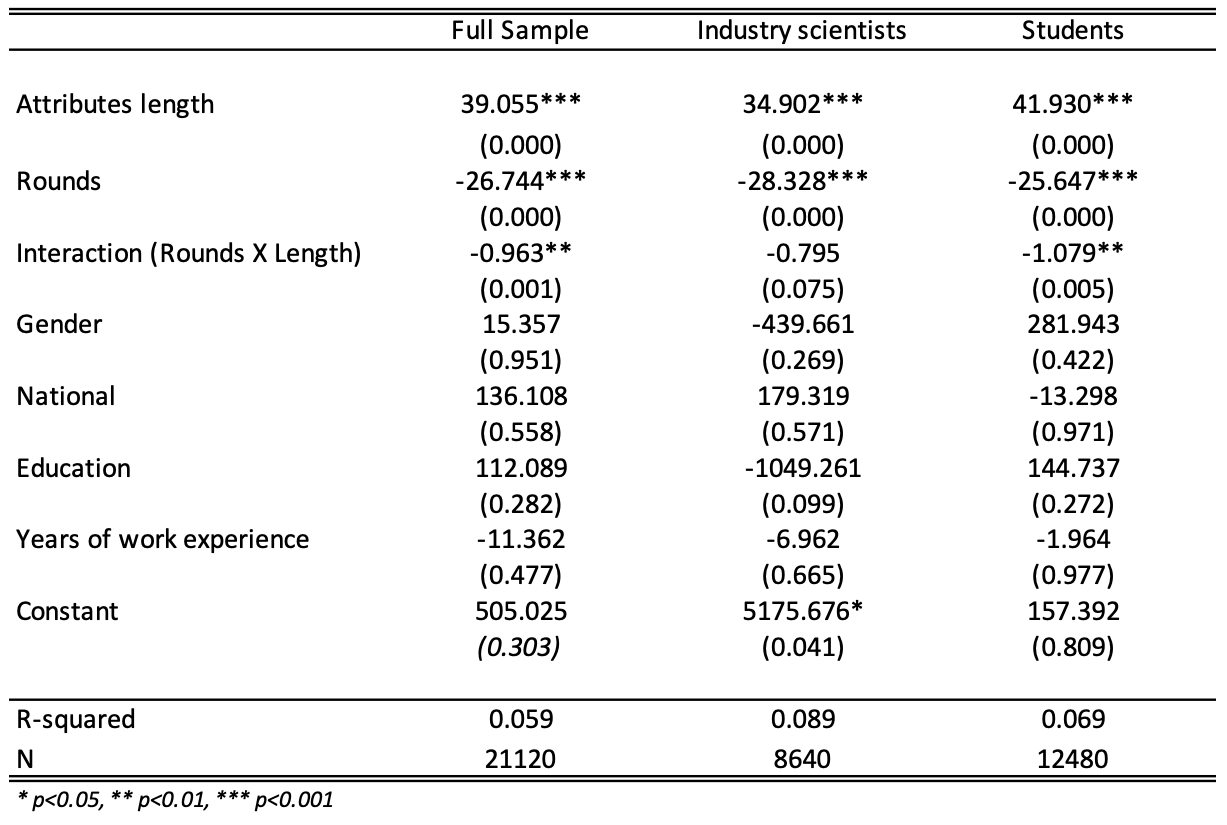

Supplement: S1 Table — Random Effects regression showing respondents’ systematic decrease in attention on the lengthy items over time. (TIF) [file pone.0278409.s002.tif]
