# Supplementary material for: Information acquisition and cognitive processes during strategic decision-making: Combining a policy-capturing study with eye-tracking data
Source: PLoS One. 2022 Dec 1;17(12):e0278409. doi: 10.1371/journal.pone.0278409 (PMC9714927; doi:10.1371/journal.pone.0278409)
Supplement: Attachment — Submitted filename: Response to reviewers.docx [file pone.0278409.s003.docx]

**Review Comments to the Author**

**Please use the space provided to explain your answers to the questions above. You may also include additional comments for the author, including concerns about dual publication, research ethics, or publication ethics. (Please upload your review as an attachment if it exceeds 20,000 characters)**

First and foremost, we would like to express our gratitude for your effort. We are extremely thankful for all the useful comments, which we believe clearly improved the quality of our paper.

We have worked through the comments and provided detailed responses and computed new results, or changed the original ones, when needed. The answers to your comments can be found below each comment in blue. Several changes have also been added to the original manuscript and these are reported in this document in italic and between quotation marks.

Thank you for the opportunity to revise and re-submit the study.

**Reviewer #1: This paper provides an interesting use of eye tracking to uncover the use of information in a policy capturing context. Unfortunately the results you found are relatively uninteresting and only marginally useful to improve policy capture. Here are a number of suggested changes that might strengthen the paper.**

**1. Relate judgment micro-processes to actual valuations. Understanding the attentional processes is important if it alters the results defined in terms of the derived policy evaluations of the attributes. Currently this seems like half a paper.**

Thanks for your comment. We appreciate your intuitive idea. The paper deliberately does not address the answers about collaboration opportunities because its focus is methodological and opening to reporting and interpretation of the actual outcomes of the policy-capturing would make the paper very lengthy and not focused on the eye-tracking application. We address the evaluation outcome of the policy-capturing study in another paper based on the same policy-capturing exercise. It was obviously a mistake from our side not to be clear about this decision. We now make the effort of being clearer about this early in the paper. The manuscript is now updated in several places to reflect this. It reads:

Page 1, abstract: *“With this study, we seek to analyze the process of answering a PC study. We do this by investigating the information acquisition and the cognitive processes behind policy-capturing, building on cognitive and attention research, and exploiting the tools of eye-tracking.”*

Page 4/5, introduction: *“We have completed a PC experiment and measured the participants’ attention processes with an eye-tracking device. The topic of the PC experiment is inter-organizational collaboration, but in the present paper, we exclusively report on the processes of answering the experiment, not on the measured drivers of inter-organizational collaboration. We have designated an entire sister paper to address the evaluation outcomes of the PC experiment [62]. We will merely connect the answers in the PC experiment to attention. Doing this, we find evidence that respondents of a policy-capturing study build cognitive models to cope with the large amount of information provided.”*

**2. Clarify your design. It is hard to understand the task used or the design across respondents. The order of the scenarios is randomized across respondents. Is the order of the attributes within scenarios randomized, or their attendant valuations?**

We apologize for not being clear enough about the task. Essentially, participants are asked to answer a survey in front of a lab computer, which is equipped with an eye-tracker. The survey is divided into two parts, which alternate randomly across respondents: 1) demographics, work-place related & attitudes questions, and 2) the policy-capturing block. While the first block includes individual direct questions to be answered on a Likert scale, the second block constitutes the actual experimental task: the policy-capturing exercise. Respondents are instructed to provide an evaluation of 30 scenarios, which are described by 16 attributes (located in the same position in each scenario) that vary in their characterization within a certain range of values (which we call *degrees* and go from “low” to “high”) in a random fashion. While the position of each attribute is the same for every scenario, its characterization, its degree, changes randomly across scenarios. All respondents eventually answer to the same 30 scenarios but each one in a different random order.

We have now clarified the task in the manuscript. It now reads:

*“In short, respondents were instructed to provide an evaluation of 30 scenarios, which are described by a list of 16 attributes (located in the same position in each scenario) that vary within a certain range of values (five degrees from “low” to “high”) in a random fashion. While the position of the attribute is the same for every scenario, its characterization (its degree) changes randomly across scenarios. All respondents eventually answer to the same 30 scenarios but each in a different random order.*

For the block structure, we tried to clarify the previous explanation. Now it reads:

*“The Qualtrics policy-capturing exercise included two instruments which alternate randomly across respondents: 1) the policy-capturing block, which consisted of an instruction page, 30 randomly ordered pages, each describing a collaboration decision scenario, and a final page where participants rank ordered the importance of the decision attributes used in the scenarios; 2) a background survey collecting information on participants’ demographics and attitudes. While the first block includes individual direct questions to be answered on a Likert scale, the second block constitutes the actual experimental task - the policy-capturing exercise.”*

**You indicated that the correlation between the 16 attributes (defined linearly 1-5) is less =/- .40 across the 30 tasks. It is surprising you could not find a design with lower correlations. There should be a design with 32 tasks that can generate 16 independent binary variables. Your example design in Figure 1A shows no evidence of any of the attributes having a low (1) rating. Is that true of all scenarios? Having 4 levels may help you find an orthogonal design. Is that what you did?**

Thank you for seeking further details on this. We adopted the randomization procedure and applied the correlation threshold from prior literature employing the policy-capturing method (Tyler et al., 1995). The randomization procedure is based on a single factor weight analysis per scenario. The example included in S1 Fig does not include any low attribute by chance, the other scenarios do. More precisely, there are three scenarios with no “low” degree included in the 30 scenarios as a result of the random characterization). Here below Fig R.1 another example for a random scenario.

Fig R.1. Additional scenario example. The picture describes the scenario outlook, indicates the distribution of the three main area of interest (attributes, degrees, answers) and provides an example of specific AOIs on which eye-tracking measures and metrics are based.

**3. Do you need both efficiency and selectivity? Conceptually, these constructs are inversely related, as a processes which are selective are likely to be less efficient. What is their correlation? The inverse similarity is evident in their statistical patterns is shown in Table 2. It might be wise to focus only on selectivity, as that is normative, provided, as you show, that one attends the attributes that are less likely to be important.**

Thanks for your comment. We agree that the measurements of efficiency and selectivity conceptually should inversely be related. We apply the concepts of efficiency and selectivity independently from each other to test different mechanisms, which we believe justifies reporting both. We define *efficiency* as the process in which decision-making progressively requires less effort to reach a resolution of a task, and *selectivity* as the process of attending on progressively smaller amounts of selected information (Meissner et al., 2016). It can be expected that, if a selection process takes place, efficiency will also increase as a consequence: less information to focus on would require less effort and, thus, an increase in efficiency. Furthermore, we use two different eye-tracking measures to study efficiency and selectivity (respectively time spent and zero-fixation attributes) and wee believe both play an important role in our analysis.

We would also like to underline that, to seek legitimization in applying eye-tracking to investigate the policy-capturing methodology, which, to the authors’ knowledge, was never done before, we decided to construct our analysis on what previous literature had done for different but comparable choice experiments. More specifically, we build on Meissner et al (2016) in relation to our efficiency and selectivity measures and on Hoeffler & Ariely (1999) for the consistency measure.

Meißner M, Musalem A, Huber J. Eye Tracking Reveals Processes That Enable Conjoint Choices to Become Increasingly Efficient with Practice. Journal of Marketing Research. 2016;ISSN:1–17.

Hoeffler S, Ariely D. Constructing stable preferences: A look into dimensions of experience and their impact on preference stability. Journal of Consumer Psychology. 1999;8(2):113–39.

We appreciate very much that you are raising this point about our measures. We have updated the manuscript to make it clearer how we establish a way of combining policy-capturing and eye-tracking based on previous work, and why we believe it is important to have both selectivity and efficiency in the analysis.

The first paragraph of the *Results* section now includes this addition:

*“We decided to opt for this approach to seek legitimization in applying eye-tracking to investigate the policy-capturing methodology, which, to the authors’ knowledge, was never done before.”*

In the same section, after defining the paper’s terminology, we add:

*“Despite efficiency and selectivity both representing a measure of attention, we believe that they play two important roles in completing our analysis respectively. On one hand, the two concepts are applied independently from each other and test different mechanisms, which we believe justifies reporting both. On the other hand, two different eye-tracking measures are employed to study efficiency and selectivity, as explained in detail in the section below.”*

**4. Play down the student vs. employed effect. The least interesting findings of this paper stem from the low difference between the processing of students vs. employed. Part of the problem may stem very small samples of respondents and from the inherent variability within categories. The only difference that matters is that it takes a little longer for the students to get used to the attributes, as one would expect. Do the two groups differ in their valuation of attributes?**

Thanks for the comment. We have included the secondary level of analysis on sample composition in the context of the ongoing conversation among experimenters on the employment of students vs. professionals. Working with students as subjects can be a strategic decision in terms of availability, reachability, and costs. That is also the reason why we believe that not finding a substantial difference in the cognitive processes behind PC is a rather positive finding in the perspective of running policy-capturing studies on strategic decision making with students as subjects. More specifically, the two groups differ in the weight they associate with certain attributes (see Table R.1). It is worth noticing that seven attributes are significant for each sample out of the 11 attributes that are significant for the overall group.

Table R.1. Policy-capturing regression table. The table shows three Fixed Effects models for the overall respondents, the student sample, and the industry sample, respectively.

We are grateful for raising this issue which we find an important contribution. We have therefore updated the motivation to clarify that it is an ongoing discussion and why that discussion is important to have. The relevant paragraph was modified and is contained in the introduction. It reads:

*“Additionally, we investigate the role of experience in mediating the relationship between the information process and the judgments to verify the reliability and appropriateness of the samples recruited. Indeed, Karren et al. [3] point out that despite being an important criterion in most research studies, few of the reviewed PC papers have analyzed the reliability of their decision makers’ judgments. This level of analysis relates to an ongoing academic debate on the use of students as experimental subjects [45-47]. Indeed, working with students as subjects, when leading to externally valid conclusions, can be a strategic decision in terms of availability, reachability, and costs. That is also the reason why we believe that not finding a substantial difference in the cognitive processes behind PC is a rather positive finding in the perspective of running policy-capturing studies on strategic decision-making with students as subjects.”*

In addition, it is important to underline that the policy-capturing study was performed during the academic year 2018/2019 as part of a funded project at the local university. Several academic outcomes, with distinct scope, level of analysis and research focus, have been developed by exploiting the data collected during that time. As a result, the evaluation of the attributes and differences among subsamples in such exercise is beyond the scope of the present paper, while being the focus of another paper about policy-capturing and the theoretical constructs behind it (as described in the manuscript in Table 1), which are not the main focus of analysis, but rather serve as a tool to investigate how the process of decision-making unfolds. We follow up to this issue at the source, by explicitly mentioning the existence of a sister paper in the manuscript. In the introduction, it now reads:

*“We have completed a PC experiment and measured the participants’ attention processes with an eye-tracking device. The topic of the PC experiment is inter-organizational collaboration, but in the present paper, we exclusively report on the processes of answering the experiment, not on the measured drivers of inter-organizational collaboration. We have designated an entire sister paper to address the evaluation outcomes of the PC experiment [62]. We will merely connect the answers in the PC experiment to attention. Doing this, we find evidence that respondents of a policy-capturing study build cognitive models to cope with the large amount of information provided.”*

**5. Do a better job with accuracy. Accuracy is key. Currently you only measure it based on the extent to which attention focuses on attributes that are valued by a respondent. A second measure is the extent to which each respondent’s choice is consistent with their judgments, assessed by either the standard error or the R-squares of within-individual regressions. Typically, that internal measure of consistency increases with round as respondents adjust their responses to be consistent with each other. A third measure of consistency is whether a respondents’ direct measures differ with that of their peers. It would be exciting to show that peer consistency increases with round, implying that they merge towards each other’s judgments over time.**

Thanks, these are very interesting points. To address the measure suggested, we average the individual R-squared of the first half of scenarios and the individual R-squared of the second half to see how much it varies. The R-squared does not show an increasing trend. Moreover, when testing the values of the R-squared for the first ten scenarios against the last ten scenarios on the overall sample, no significant effect is detected (p=0.507). When splitting the sample by students and industry scientists, no significant effect is found either, which suggest that experience seems not to have an impact on the levels of R-squared of the individual regressions. This result suggests that the individual consistency does not develop over the time course of the experiment, but it remains stable throughout.

In relation to the other idea on peer effects, we investigate the standard deviation of the individual R-squared and do not find a clear trend (see Fig R.2); splitting the sample across students and professionals leads to similar results. This suggest that the answer does not become systematically more similar to the answers of peers over the course of the experiment.

Fig R.2. Accuracy. Plot of standard deviation of individual R-squared for the overall sample (Panel A) and for the split sample (Panel B).

*Panel A – Overall sample	Panel B – Split Sample*

We added these results at the end of the *Experience and consistency* subsection, which now reads the following addition:

*“Another internal measure of consistency is the extent to which each respondent’s choice is consistent with their judgement by the means of the R-squared of their individual regressions. To address this, we average the individual R-squared of the first half of scenarios and the individual R-squared of the second half to see how much it varies. The R-squared does not show an increasing trend. Moreover, when testing the values of the R-squared for the first ten scenarios against the last ten scenarios on the overall sample, no significant effect is detected (p=0.507). When splitting the sample by students and industry scientists, no significant effect is found either, which suggest that experience seems not to have an impact on the levels of R-squared of the individual regressions. This result suggests that the individual consistency does not develop over the time course of the experiment, but it remains stable throughout.”*

**6. Separate aggregate from individual analysis. It would be helpful to display a regression pooling all respondents together. That enables tests of shared strategies across individual. Then run regressions within individuals. Those individual tests can assess the extent to which individuals used different strategies. Below are four potentially valuable tests.**

Thank you for the four insightful suggestions. We have interpreted point 6) as an introduction to the following 4 points. For the sake of clarity, we have split point 9) into two different comments 9) and 10). We therefore address the suggested strategies here below and we integrate the original manuscript when new, relevant insights were found.

**7. Test the impact of attributes near the top. Check to see whether most searches move from top to bottom. If that occurs, there may be enough information for a reasonable evaluation to be made for items early in the process, thus limiting time spent at a low accuracy cost.**

Thank you for the suggestion.

1. We find indeed a top-to-bottom trend in terms of the sequence of attention. When looking at the average Time to First Fixation (TTFF) for the 8 attributes highest on the display of each scenario and the lowest 8 attributes, we find a significant difference: the average time to first fixation for the highest 8 attributes is 23.665, while the average time to first fixation for the last 8 attributes is 31.103 (p<0.001).
2. Moreover, also when we look into the time spent on the highest 8 attributes compared to the lowest 8 attributes, we find a difference that seems to suggest a downward trend. When splitting for the first and the last 10 scenarios, such difference is less pronounced in the first ten scenarios compared to the last ten scenario.

We have updated the manuscript to reflect this finding. It reads:

*“We find that participants on average attend to the first half of the attributes graphically displayed on the screen faster than the second half displayed on the lower part of the screen (time to first fixation is on average respectively 23.66 and 31.10; p<0.001), suggest that a top-down process applies.”*

**8. Test the impact of the lengths of the attribute descriptions. With experience, the time taken focused on an attribute may drop because respondents can recognize its value without having to reread the sentence. This drop is more likely for long descriptions.**

Thank you for the comment, the length of the attribute descriptions is indeed an interesting feature to test. In order to take the attribute length into account, we have created a variable which signals the number of words that each attribute is described with (e.g., the first attribute corresponding to 14, the second one with 18, etc.). To test if, with experience, the time drops because of sentence recognition, we regress the following regression with a full sample and dividing it by professionals and students, which somehow replicates and adds on Table 2 included in the original manuscript. The dependent variable is time spent per attributes, while we include the order of scenarios “Rounds” and the number of words “Attributes length” as independent variable; we then interact the two independent variables to check the effect of attributes length while the scenarios succeed one another. In order to allow for the interaction between the variable order and the attributes length, it is important to underline that all observations needed to be included. Therefore, compared to the original Table 2, which was built at the scenario level and included N=1320 observations as a result of 30 scenarios X 44 subjects, the coefficients of Table R.2 seem inflated due to the sample size (N=21120 as a result of 30 scenarios X 44 subjects X 16 attributes per scenario). Table R.2 shows that respondents spend systematically less time on the lengthy items over time. When splitting the sample into industry scientists and students, we notice that the effect is driven by students.

Table R.2. Attribute Length and Rounds. Random Effects regressions of attributes length, order of scenarios and their interaction on time spent per attribute, divided by samples.

We integrate the latter finding in our manuscript in the second last paragraph at the end of the subsection *Experience and Efficiency*; it reads:

*“An additional interesting feature to test in the context of efficiency is the length of the wording of each attribute description. Thus, a variable indicating the number of words that each attribute is described with (e.g., the first attribute corresponding to 14, the second one with 18, etc.) was created. To investigate the role of the order of scenarios and the attributes length simultaneously, we have run an additional Random Effects regression, which replicates and adds on Table 2, in S1 Table. S1 Table shows that respondents spend systematically less time on the lengthy items over time. When splitting the sample into industry scientists and students, we notice that the effect is driven by students.”*

Therefore, please note that Table R.2 was added to the supplementary materials of the manuscript as S1 Table.

**9. Test whether some respondents may be ignoring the attribute labels and simply averaging degree information. That shortcut is even easier if one evaluates the scenario by its number of 5’s. More complex but almost as easy is the number of 4’s or 5’s, or the number of 5’s minus the number of 1’s. It should be possible to measure saccades down the column to see if they are scanning down to look for other similar ratings.**

That is an interesting take that we have not considered before, thank you for raising it. To address this suggestion and check if respondents’ attention and decisions are not driven by the attributes but rather by the degree values, we plot the TTFF for each value the attribute takes on.

Fig R.3 represents the average values of TTFF by degree values. No significant difference at the aggregate level is detected and, no matter what the value of the degree is, the correspondent attribute catches the attention at around 32 seconds (specifically, at 32.29 for value=1; 32.242 for value=2; 32.56 for value=3; 32.06 for value=4; 32.06 for value=5).

Fig R.3. Time to First Fixation for degree value. The graph shows the average TTFF for different values of the degrees in Panel A and split for all 30 scenarios in Panel B. Note that in column 1, for example, there are included all the areas of interest on degrees that assume the value 1.

*Panel A – on average	Panel B – for each scenario*

When looking at each scenario, which has the same degree value coding for all respondents, the distribution of TTFF looks fairly homogenous. Please note that the missing columns correspond to scenarios where the degrees never assume that specific value for which the column is missing.

In sum, higher values of degrees do not to attract attention before lower values. We find the same conclusion when analyzing the participants’ reported importance of each attribute instead of TFFF.

Finally, we look at the relationship between the attributes with more 5s and 4s degrees and their evaluation from the stated preferences (Ranked data) and the significance levels of attributes (PC data). Our data do not show higher ranked importance of the attributes with more 5s and 4s. The same picture emerges from the PC data: when looking into the probability of an attribute being significant (with a *probit* regression), a variable counting how many 5s that each attribute has does not show any significance. The same occurs for 4s and for 5s and 4s together.

Therefore, in sum, it seems that respondents’ evaluations are not anchored by the number of 5s and 4s degrees that an attribute has.

**10. Finally, test whether respondents are first exploring important attributes and then examining their ratings. A number of respondents initially examine the attributes top down and then examine the degree for each. If so, you will find attribute-to-degree moves but few in reverse. The selectivity then comes in by moving immediately to a few important attributes.**

Thank you for the contribution, this is a very interesting angle. To test this, we focused on the variable *Time to First Fixation* (TTFF) in our dataset and we analyzed it for attributes and degrees separately. First, for each respondent, we take the most important and the least important attributes, as stated in the Ranked Data, in which subjects are asked to rank the attributes from most to least important, and we check how the difference between TTFF for attributes and TTFF for degrees develops.

Fig R.4 illustrates the outcome introduced above: the bar chart shows the average Time to First Fixation (in seconds) for the most important and least important attributes and degrees, respectively. It shows that there is no trend in faster attending to the most important items compared to the least important items, both in the case of attributes and degrees. Despite expecting a difference in how the attributes’ importance impacts the sequence of attention, the two timings seem to replicate quite equally between the most and least important attributes and degrees, with a clear prevalence of attributes (34.9 s on average for the most important attributes; 21.8 s for the least important attributes; 43.9 s on average for the most important degrees; 23.9 s for the least important degrees) being looked at first: a lower TTFF indicates that a certain item is looked at earlier than another one with a higher TTFF. A t-test confirms that in both situations the difference between TTFF on attributes and degrees keeps being significant (t = 86.9473, p<0.0001 for the most important attributes; t = 37.1092, p<0.0001 for the least important attributes), which shows that the importance placed on the item does not play a key role in terms of attentional sequence.

Fig R.4. Time to First Fixation for attribute importance. Bar graph for Time to First Fixation in seconds for most important items and least important items for both attributes and degrees.

Secondly, instead of across attributes and degrees within the most or least important items, we now look into the difference occurring among early and later scenarios. We therefore split the analysis for the first ten scenarios and the last ten scenarios. Fig R.5 shows how the difference in TTFF decreases between attributes and degrees for later scenarios. This can be interpreted as a way to measure whether respondents initially examine the attributes top-down and then examine the degrees to eventually becoming more efficient in identifying the attributes of interest and checking the related degree straight away without respecting a similar sequence than in earlier scenarios. However, when testing for the difference between the most important and least important items, during the first ten scenario and the last ten scenarios, all differences result significant indistinctly, which is not aligned to what was expected by the comment.

Fig R.5. Time to First Fixation for all. Bar graph for Time to First Fixation in seconds for all items, divided by the first and last ten scenarios.

Overall, people become faster and decrease their TTFF (average TTFF for attributes in the first ten scenarios = 34.9; average TTFF for degrees in the first ten scenarios = 43.9; average TTFF for attributes in the last ten scenarios = 21.8; average TTFF for degrees in the last ten scenarios = 23.9). However, while the difference between degrees and attributes seems to close in for later scenarios (suggesting that the process over time is that people check the attribute description before their characterization in degrees in the initial rounds and the TTFF between first and later scenarios becomes more similar in the last rounds), the statistical findings show that there is no strong tendency since both t-tests between TTFF to attributes and to degrees are significant (ttest for first ten scenarios: t = 86.9473, p<0.0001; ttest for last ten: t = 37.1092, p<0.0001).

**Reviewer #2: The authors present an analysis of eye movements and decisions during a policy capturing (PC) experiment in which participants rated the potential of 30 possible academic collaboration opportunities. Looking at the literature, this seems to be the first, or one of the first studies to use eye tracking to study decision strategies in a policy capturing paradigm. The paradigm, however, resembles the discrete choice experiment (DCE) to quite an extent and in the context of this paradigm, eye tracking has been used extensively. The authors refer to the DCE eye tracking papers, but I think better use could be made between the overlap, and a clearer discussion of the differences as well. Because this is (one of) the first paper(s) studying eye movements in PC, I think the paper makes a significant contribution. That said, I would have several comments that I think need addressing before I can recommend publication.

(1) The authors indicate that they cannot share data due to sensitive content. This is somewhat surprising, because the combination of expert/novice, fixation information, and decisions for the various policies does not allow for tracing the data back to participants. I therefore think the data should be made available (using participant numbers). With these data, I think the exact contents of the presented policies should also be made available. Any demographics such as age and gender can be omitted from the shared data, as they are not used for the analysis and could lead to identification of the participants.**

We do believe data sharing is fundamental for the sake of transparency, replicability, and clarity. The data were now anonymized and made available on the platform *Figshare* at https://doi.org/10.6084/m9.figshare.19753417.v1. The demographics are used for Hypothesis 1 and so were included in the dataset.

Pizzo A, Fosgaard T, Tyler B, Beukel K. Information acquisition and cognitive processes during strategic decision-making: combining a policy-capturing study with eye-tracking data. Figshare Dataset. 2022. <https://doi.org/10.6084/m9.figshare.19753417.v1>

We shared the location of our dataset also in the manuscript, which now reads:

*“The complete dataset, without any identification of the participants, is posted on the Figshare platform [115].”*

We apologize if the exact content of the policy-capturing instrument needed to be more readily available. It is currently available as the Supplementary Material.

**(2) Various plots are shown as bar plots. I think it would be better to use line plots here, using error bars to indicate the variability across participants.**

Thank you for the suggestion. Here below you may find a new version of Fig 2 and Fig 5 with the new line plots and error bars as you have suggested. We appreciate your comment since it gave us the opportunity to show our descriptive results with higher efficacy. We have substituted the figures in the paper.

Fig 2 (new version). Zero-fixations attributes and degrees. Distribution of zero-fixations attributes over the succeeding scenarios for attributes (Panel A) and degrees (Panel B). The error bars are depicted in dark red.

*Panel A - Attributes	Panel B - Degrees*

Fig 5 (new version). Zero-fixations attributes and degrees by sample. Distribution of zero-fixations attributes over the succeeding of scenarios for attributes (Panel A & B) and degrees (Panel C & D) by sample composition. The error bars are depicted in dark red**.**

*Panel A - Industry	Panel B - Students*

*Panel C - Industry	Panel D - Students*

**(3) As indicated, the policy capturing (PC) paradigm seems to be quite similar to discrete choice experiments (DCEs), for which there is an extensive literature on whether or not eye movements may reveal cognitive processing. I think you could do more with this literature in your introduction, for example, to make predictions, and in your discussion, to explain why the data are the way that they are. For example, the conclusion from the DCE literature seems to be that participants follow a fairly standard trajectory through the information presented on the screen (very much top-to-bottom, left-to-right), but that eye movements provide relatively little information about cognitive processing (which would be in line with your observations that experts and novices show similar eye movement patterns). Likewise, an important topic in the eye tracking DCE literature is attribute non-attendance (ANA), where three ways of measuring ANA have been identified: stated ANA, inferred ANA and visual ANA (the latter on the basis of eye movements). Studies have suggested that these three may not always be aligned. This could be in contrast with your finding that participants are well able to indicate which attributes are important for their decisions.**

Thanks for bringing up these comments, which we really appreciate.

We have now mentioned how the combined choice modelling and attention research help shape our hypotheses in the introduction (it is further discussed in the *Hypotheses* section). On page 4, it reads:

*“Prior work at the intersection of attention and choice modelling contributes to our four hypotheses about how participants complete the PC experiment. The hypotheses, further discussed below, are that respondents become more selective, more consistent, and more efficient over the course of the experiment, and finally that experience also affects these cognitive processes.”*

We have also extended the discussion to return to the inspiration from discrete choice experiments. It reads:

*“Policy-capturing experiments share many similarities with studies in choice modelling, and our study draw on insights from research at the intersection of choice-modelling and eye-tracking, when building its hypotheses [48,49]. It is interesting to note that despite the similarities with policy-capturing experiments, our study produces somewhat different outcomes. Where we find, through attention, that our participants develop cognitive models to make their decisions, evidence from discrete-choice experiments and eye-tracking combined does not find documentation of similar cognitive model being developed, but rather that participants follow systematic search patterns in their information acquisition [52,116]. A potential reason for this difference could be that policy-capturing studies include more extensive amounts of information in each scenario to be considered compared to choice-modelling studies, resulting in a larger need for developing cognitive models to cope with the situation. Another noteworthy difference relates to areas or attributes of a choice situation that are not attended to: attribute non-attendance (ANA). Whereas the eye-tracking discrete-choice experiments find mixed results about how visual ANA, stated ANA, and inferred ANA relate [83,117], we find that our participants are generally able to state and attend to the attributes they put weight on in their policy assessments. Despite the differences that do exist across policy-capturing and discrete choice experiments, we believe that not only does our study benefit from bridging evidence from eye-tracking discrete-choice modelling research, but that such approach should represent a general inspiration for future studies to integrate insights from related disciplines.”*

**(4) That said the policy capture method seems to differ from the DCE method in that many more attributes are shown (typically DCEs have around 4 to 7 attributes), that ratings are collected (rather than choices between options), and that no systematic method seems to be applied to decide which attributes and which levels to present to participants (in DCEs a method called d-efficient designs are used to optimally select attribute levels across choice tasks). I think it would be important to contrast the two methods in your work, and explain what can be learned from the DCE literature for PC and what cannot.**

Thank for the comment. It is a really good point. We have been so focused at explaining how the methods are similar that we missed the obvious fact that differences exist. Thanks for pointing this out. We have updated the manuscript to reflect this. It reads:

*“Policy-capturing and choice modelling methods share many similarities, as emphasized by Aiman-Smith et al. [9], and we therefore build our analysis upon the experimental approaches previously applied on choice modelling studies, such as in Meissner et al. [48] and in Hoeffler et al. [49]. It is important to note that differences do exist between the methods, as such the number of attributes the respondents are exposed to, whether the extracted rating is continuous or not, and whether the attribute selection is theoretically driven or empirically driven.”*

**(5) I think the discussion could be more like a discussion. I would very much like to see a comparison between the present results and past findings (there is quite a bit of literature on eye movements for discrete choice experiments), discussions of any differences with past findings, how results fit in existing theories (such as the eye-mind hypothesis mentioned in the introduction) and a discussion of possible limitations of the study and future directions.**

Thanks for the comment, and we agree that it is a good idea to broaden the *Discussion* section somewhat. As mentioned above, we have added the following on the comparison between our findings and findings of the discrete choice literature.

*“Policy-capturing experiments share many similarities with studies in choice modelling, and our study draw on insights from research at the intersection of choice-modelling and eye-tracking, when building its hypotheses [48,49]. It is interesting to note that despite the similarities with policy-capturing experiments, our study produces somewhat different outcomes. Where we find, through attention, that our participants develop cognitive models to make their decisions, evidence from discrete-choice experiments and eye-tracking combined does not find documentation of similar cognitive model being developed, but rather that participants follow systematic search patterns in their information acquisition [52,116]. A potential reason for this difference could be that policy-capturing studies include more extensive amounts of information in each scenario to be considered compared to choice-modelling studies, resulting in a larger need for developing cognitive models to cope with the situation. Another noteworthy difference relates to areas or attributes of a choice situation that are not attended to: attribute non-attendance (ANA). Whereas the eye-tracking discrete-choice experiments find mixed results about how visual ANA, stated ANA, and inferred ANA relate [83,117], we find that our participants are generally able to state and attend to the attributes they put weight on in their policy assessments. Despite the differences that do exist across policy-capturing and discrete choice experiments, we believe that not only does our study benefit from bridging evidence from eye-tracking discrete-choice modelling research, but that such approach should represent a general inspiration for future studies to integrate insights from related disciplines.”*

In addition, we added the following discussion on theories, limitations, and future directions.

*“Our findings can be viewed as an application of the eye-mind hypothesis. Our participants tend to attend more to the elements which are both revealed to be important for their answers to the policy-capturing exercise but also rated by themselves as important, suggesting that the eye-mind hypothesis fits also with our applied setting.*

*Finally, a fundamental methodological contribution of our study is to showcase the potential gains of combining an existing empirical methodology with attention evidence. Such combination allows researchers to go beyond the assumption of a particular cognitive process and to explicitly map the process instead. Methods, such as vignette studies, are often built on more or less explicit cognitive assumptions. We show that a promising way to conduct future research is to directly investigate the information acquisition and cognitive processes of such instruments through attention measures.*

*Our study is the first to combine eye-tracking and policy-capturing. Although providing novel insights on the attention process during a policy-capturing study, a number of limitations naturally applies. First, following the standard choice design in policy-capturing methods, we keep the order of the displayed items fixed. It would be interesting to randomly vary the order to create a causal relation on micro processes such as center bias and top-down processes. Second, we fixate the information amount of each scenario, following the standard method specification. Randomly varying the amount of information would allow to determine whether using more information indeed results in participants creating heuristics, as assumed in policy-capturing studies.*

*We hope future studies will appreciate our initial findings that participants do build cognitive shortcuts to complete the policy-capturing exercise, but also continue to explore more how and when those shortcuts develop.”*

**(6) I found it difficult to understand how the eye movement data were recorded and analysed. The survey seems to be presented in Qualtrics, which seems to be an online survey tool. Eye movements seem to be collected with a Tobii T60 eye tracker. How was the onset of each question aligned with the eye movements?**

Thanks for raising this problem. The *Tobii T60* eye-tracker is operated on the *iMotions* software platform. iMotions combines data collection from the survey and eye-tracker in a synchronized way. iMotions automatically segmented eye-tracking variables (e.g., number of fixations, amount of time spent, number of gazes, time to first fixation, hit time, number of revisit fixation duration). In addition, Qualtrics is an online software tool which we employed for collecting the questionnaire answers. The fact that the questionnaire was online does not have an impact in our setting. Other survey tools could have been used but, due to the specific randomization needs of the experiment, Qualtrics seemed an appropriate and efficient tool to use. The eye-tracking data were stored on iMotions in the lab computer, while the policy-capturing decision data were stored online on Qualtrics and eventually downloaded. The two datasets were separately analyzed, then made compatible and merged for common analysis.

In practice, when the respondent was in the right position in front of the lab computer and ready to answer, the screen started showing the questionnaire and the participant started answering it. Thanks to the online link plug-in function of iMotions, it was possible to overlap and temporally align what was showcased on the computer screen and the data collected by the eye-tracker.

We have updated the manuscript to avoid misunderstanding about this. It now reads:

*“In relation to data collection, the software iMotions combined data from the survey and from the eye-tracker in synchro. iMotions automatically segmented eye-tracking variables (e.g., number of fixations, amount of time spent, number of gazes, time to first fixation, hit time, number of revisit fixation duration). The eye-tracking data were stored on iMotions in the lab computer, while the policy-capturing decision data were stored online on Qualtrics and eventually downloaded. The two datasets were separately analyzed, then made compatible and merged for common analysis. Thanks to the online link plug-in function of iMotions, it was possible to overlap and temporally align what was showcased on the computer screen and the data collected by the eye-tracker.”*

**(7) For the fixations, did you make use of the automatic segmentation method of the T60 eye track to separate samples into fixations and saccades?**

Yes, the segmentation of the different eye-tracking variables was automatic and calculated by the eye-tracking software employed to collect the data (*iMotions A/S*, version 7.1). We did not define a fixation differently from what the iMotions software was programmed to do, nor set a different threshold for the eye-tracking variables defined for data collection.

**(8) I’m a bit worried about line 208 where you indicate to only use fixations of at least 200ms. That is quite a high threshold. I have seen thresholds of 80ms being used, but looking at the fixation distributions provided by Rayner in his articles and books, I think you may be missing quite a substantial number of fixations in your analysis by using a 200ms cut-off duration.**

Thank you for this comment. This is an oversight of ours. We had included such specification as an effort to provide a short, technical definition of fixations as explicitly reported in Meissner et al., which is one of our two main references from an analytical standpoint. However, it is a confusing sentence since we explain in detail the terminology and technicalities related to the eye-tracker in the *Materials and Methods* section. We now explicitly state the ms threshold for fixations applied in the study, which is 100 ms. Such threshold was not deliberately decided by the authors but employed as part of the data segmentation of the eye-tracking software used for the study (*iMotions A/S*, version 7.1). In iMotions, if the duration of a fixation is less than 100 ms, it gets discarded. Only fixations candidates that are longer than 100 ms count as a fixation. We apologize for the confusion. We have updated the manuscript, which now reads:

*“The duration threshold in milliseconds (ms) to identify a fixation is 100 ms. Such threshold was not deliberately decided by the authors but employed as part of the data segmentation of the eye-tracking software used for the study (iMotions A/S, version 7.1). In iMotions, if the duration of a fixation is less than 100 ms, it gets discarded. Only fixations candidates that are longer than 100 ms count as a fixation.”*

**(9) In line 255 you indicate to follow the method by Hitt et al and Tyler et al. I think it would be important to briefly explain what these methods involve.**

Thanks. It is a very important point also in relation to other comments on the survey design. The two papers mentioned are considered in the organizational and strategic management literature as the main references in relation to the establishment of the policy-capturing methodology used in our study.

We now include this explanation in the manuscript. It reads:

*“More specifically, Hitt et al. [6] were the authors who in 1979 developed the specific design of the policy-capturing (PC) questionnaire applied in several papers thereafter. The specificity of this version of PC concerns the number of the repetition of the scenarios, the number of the attributes included and repeated in each scenario, the randomization of the order in which the scenarios are administered to the respondents versus the iteration of the same orders of the attributes (randomized only once during the design phase). The application of the policy-capturing methodology, as develop by Hitt et al. [6], while providing a close reference to prior and well-established literature, made us preserve the key feature of the questionnaire design”*

**(10) Part of the participants seem to have participated online (no eye tracking) and part took part in the eye tracker. What is unclear to me is how these data were analysed. Were eye tracking outcomes based on just the eye movement participants while the decision data were based on all participants? What was done for analyses that involved the combination of decisions and eye tracking data?**

Thank you for the comment. The analysis of this study only includes participants who took part of the version of the Policy-capturing exercise in the eye-tracking setting. We use the combination of the decisions and eye-tracking data of the same respondents. All respondents have taken part to the study in similar conditions.

We added a specification in the manuscript (in the *Sample* subsection). It reads:

*“Both subsamples took the policy-capturing exercise in the eye-tracking setting.”*

**(11) The time spent (often called dwell times) seem to be reported in seconds. This may be problematic, because some participants take a long time to decide, while others take less (so those who spend more time on each task contribute more strongly to the average data). Time spent on tasks also decreases over time, and therefore tasks early in the sequence contribute more than later tasks. I would strongly recommend to also consider time spent as a percentage of the total trial duration, to reduce such length effects.**

This is a very good point which we did not include in our measurements. Thank you for raising it. In order to address it, we built an individual measure of time spent on each scenario as a proportion of the total time spent on all 30 scenarios for each participant. We then checked how this new measure would change our results. To do so, we replicated Fig 1 in Fig R.6: the course of the graph corresponds to the previous version of the figure on average time spent in seconds. The only difference is that, differently from the original version of Fig 1, the degrees average level seems higher in relation to the attributes’ tail of the curve. We have also replicated the results displayed in the manuscript as Table 2 in Table R.4 and the significance levels hold, despite a drop in coefficients due to the different scaling of the dependent variable.

Fig R.6. Time spent as proportion of total. Pattern of attention measured as an average of the individual proportion of time spent for each scenario out of the cumulative time spent for all scenario for attributes (Panel A) and degrees (Panel B).

*Panel A – Attributes	Panel B - Degrees*

Table R.4. Time spent as proportion of total (regression). Random effects regression models for both attributes and degrees covering hypothesis 1.1 and hypothesis 1.2 (p-values within brackets).

It is indeed a positive outcome the fact that we do not find any difference to our own measures, but we highly evaluate a good sensitivity check, like the present measure of relative time spent instead of the absolute time spent in seconds that is included in the paper. We thank you for the suggestion. We therefore decided to keep the measure of time spent as it is in the paper, which relates to previous literature (i.e., Hoeffler et al. [49]), and address this sensitivity exercise in a comment on our *Efficiency* subsection, which now reads:

*“To have a more relative measure of attention and as a sensitivity check, we replicated the same analysis as above (Fig 1 and Table 2) with an individual measure of time spent on attributes and degrees as the proportion of the total time spent on all 30 scenarios for each participant: the same results hold.”*

**(12) It is unclear to me what the purpose of the pilot was. How much was changed to the study protocol after the pilot?**

Thank you for raising this question. This study was the first lab experience for the co-author who implemented its application. Therefore, we planned to have a pilot study to stress test the understandability of the survey, built on the Qualtrics software, and, above all, to ensure a smooth procedure in the eye-tracking lab. After having performed the pilot, we identified and solved practical issues: for instance, we adjusted the scheduling of respondents based on the actual participation times among the pilot subjects, we designated a specific waiting room next to the lab, we increased the physical signs indicating the location of the lab facility at the University building, we increased our efficiency in the verbal explanation prior to the experiment. In addition, we were also able to perfectionate the standardization of the in-person protocol that we followed, which consisted in the repetition of the same verbal interaction with participants. Finally, from the data analysis of the pilot study, we identified a coding mistake in the randomization of the scenarios in Qualtrics and we were luckily able to fix it.

**(13) Please make sure that all statistics are reported with the same number of digits (sometimes there are too many).**

We agree that the lack of consistency in the number of digits included was confusing, now it is fixed. Statistical coefficients and p-values include three digits after the dot, while descriptive means only have two. Thank you for the spot-on comment.

**(14) Please have the paper proof-read one further time before resubmiting.**

Thank you for the suggestion. We have proof-read the paper again and spotted some missed typos.
