# Supplementary material for: Information acquisition and cognitive processes during strategic decision-making: Combining a policy-capturing study with eye-tracking data
Source: PLoS One. 2022 Dec 1;17(12):e0278409. doi: 10.1371/journal.pone.0278409 (PMC9714927; doi:10.1371/journal.pone.0278409)
Supplement: Attachment — Submitted filename: PONE22 3792 tracking judgments.docx [file pone.0278409.s004.docx]

Thank you for your careful responses to the questions in the first draft. I believe there are some important isssues that need to be resolved for this to be an important paper that helps understand multi-attribute scenario judgments.

**Initial Reactions:**

1. Figures 1 and 4. Figure 1 shows time by task order Perhaps convert both to log scale. Double log learning curves tend to be quite linear. Figure 4 provides very little information not found in Figure 1. You might simply present Figure 4 first and then table 4 showing differences in time spent differ little by professionals and students.
2. Figure 2; Attributes-not-viewed. Are these relevant differences? It might be more meaningful to provide the % of information viewed per attribute rather than a binary scale of teh proportion of attributes viewed vs. not viewed. .
3. Figure 5 shows zero fixation attributes and degree by sample. Consider dropping Figure 5 since there are visual differences that seem apparent but are not statistically reliable. You could simply include student vs. Professional as an analysis table.
4. Figure R2 does not make sense as the three graphs are virtually identical.

**Suggested changes:**

1. Play down the distinction between attributes and degrees. One possibility is that respondents may simply average the degrees for each scenario, but that would result in null differences between the attributes. A good normative rule specifies that the more important the attribute the more valuable it is to assess its degree. Generally, it is wasteful to spend relevant time on an attribute without considering its degree. If so, then there should be few examples where a person fixates on an important attribute without noting its scale. To test that calculate the percent of attributes that are viewed without examining its degree, and the number of degrees sampled without viewing its attribute. Those may be a small percent (say 20%) and should decrease with round or in cases where substantial time is spent on either (say > 500 ms). Below I suggest an analysis that merges attribute and degree time into one measure of attribute attention.
2. Play down the selectivity metric from zero fixations. The problem with eye tracking is that many fixations occur randomly or to they are briefly noted and then ignored. On Table 4 the standard errors from the selectivity measures are substantially larger than the efficiency measures. One way to fix that is to define a higher cutoff for what makes a fixation, say at 500 ms. The other way is to simply focus more on what people spend more time on rather than not. Put differently, the important measure is the total time of fixations rather than the distinction between attributes with no fixations vs. some.
3. Make a run of 21,000 observations similar to what you did in R2. The initial large estimates and small error terms in R2 lack credibility. Perhaps the measure is in milliseconds? The small errors may come from assuming that the errors within persons and tasks are uncorrelated with each other. If so, better results may come from the following analysis:
4. Predict attribute plus degree seconds with the following independent variables: fixed constants for each of the 16 attributes, fixed constants for each of the 43 respondents, continuous measures of attribute length, individual’s ranking for each attribute, and rounds. The main effects will show the relative impact of attribute length, individual ranking, and rounds. Then interactions between rankings, attribute lengths, and rounds (zero centered) will indicate whether rankings become more predictive of decision time with rounds, whether attribute length becomes less predictive with rounds, and whether attribute rank and length and ranking interact to predict attribute time. The idea is to identify the important factors that alter decision time.
