# Supplementary material for: Information acquisition and cognitive processes during strategic decision-making: Combining a policy-capturing study with eye-tracking data
Source: PLoS One. 2022 Dec 1;17(12):e0278409. doi: 10.1371/journal.pone.0278409 (PMC9714927; doi:10.1371/journal.pone.0278409)
Supplement: Attachment — Submitted filename: Response to reviewers_SECOND REVISION_01102022.docx [file pone.0278409.s005.docx]

**Dear Editor, Dear Reviewers,**

**Thanks for the opportunity to resubmit our paper. We are very thankful for your constructive comments and suggestions which are all well-taken. Below, we are responding to all the comments and are highlighting the changes made in the manuscript as a reaction to the comments (our answers are in blue). Overall, the comments and the suggested changes have, in our opinion, improved the manuscript substantially. We would like to mention that we found a few mistakes in the first “response to reviewers” document. For that reason, we are re-submitting the first reply document (with the corrections), along with this second reply document.**

**Reviewer 1:**

Thank you for your careful responses to the questions in the first draft. I believe there are some important issues that need to be resolved for this to be an important paper that helps understand multi-attribute scenario judgments.

**Initial Reactions:**

1. Figures 1 and 4. Figure 1 shows time by task order. Perhaps convert both to log scale. Double log learning curves tend to be quite linear. Figure 4 provides very little information not found in Figure 1. You might simply present Figure 4 first and then table 4 showing differences in time spent differ little by professionals and students.

Thank you for the suggestion. We have followed your suggestion by converting the Average Time Spent of the following exhibit into the logarithmic form. In the Manuscript, we keep Figure 4, now renamed as Figure 1, and we take away the old version of Figure 1.

The old version of Figure 4 is dropped, Figures 5 and 6 are now Figure 4 and Figure 5 (see additional change in answer 3). Figure 1 includes the distinction across the professional and the student sample, and the text was updated as follows:

“The development of attention over time is shown in Fig 1, which also includes the split between Professionals and Students.”

**Fig 1. Attention.** The pattern of attention; time spent (seconds), log transformed, for attributes (Panel A) and degrees (Panel B) over the 30 repeated collaboration scenarios, by professionals and students.

2. Figure 2. Attributes-not-viewed. Are these relevant differences? It might be more meaningful to provide the % of information viewed per attribute rather than a binary scale of the proportion of attributes viewed vs. not viewed.

Thanks for the comments, which are well-taken. Unfortunately, we do not have access to the original raw data from the study due to a change of institutional affiliation. For this reason, we cannot reshape our Areas of Interest (AOIs) and consequently we cannot retrieve data on the percentage of information viewed per attribute (i.e., measure how many words of each item have been viewed). We would also be concerned with the data reliability as the precision the applied eye-tracker is not high enough to create AOIs for each word.

We like the suggestion though, and have created alternative measures, displayed below, which are showing how many average fixations our participants have across the attributes and the degrees.

**Figure 2-bis. Fixations.** Additional Panels on the average number of fixations of all attributes per each scenario for both degrees and attributes.

The figure illustrates the average intensity of fixation per scenario actually looked at. The trend in both panels resembles, as one can expect, the course of the time spent on each scenario, as addressed in the first hypothesis. We make a comment on this in the manuscript now. It reads as follows:

“As a contrast to the zero-fixations, we also analysis what is in fact fixated on. A paired t-test comparing the average fixations of the first ten scenarios with average fixations in the last ten scenarios finds a significant difference for attributes (p<0.0001), with 28.8 fewer fixations on average. For the degrees, the difference is also significant (p= 0.0018), with 3.1 fewer average fixations.”

3. Figure 5 shows zero-fixation attributes and degree by sample. Consider dropping Figure 5 since there are visual differences that seem apparent but are not statistically reliable. You could simply include student vs. Professional as an analysis table.

Thanks for the suggestion. We have dropped Figure 5 and kept the discussion about the subgroup’s tests in the manuscript. The exhibit related to this result is still Table 3. Because Figure 5 was dropped, the exhibit included in the following section Experience and Consistency is now named Figure 4.

4. Figure R2 does not make sense as the three graphs are virtually identical.

There was indeed a mistake on the exhibits we had uploaded for Figure R2 Panel A and B. We are grateful you have spotted it and we have now substituted them. We find that the updated exhibit is relevant and have decide the keep it in the text.

Fig R1.2. Accuracy. Plot of standard deviation of individual R-squared for the overall sample (Panel A) and for the split sample (Panel B).

*Panel A – Overall sample	Panel B – Split Sample*

**Suggested changes:**

1. Play down the distinction between attributes and degrees. One possibility is that respondents may simply average the degrees for each scenario, but that would result in null differences between the attributes. A good normative rule specifies that the more important the attribute the more valuable it is to assess its degree. Generally, it is wasteful to spend relevant time on an attribute without considering its degree. If so, then there should be few examples where a person fixates on an important attribute without noting its scale. To test that calculate the percent of attributes that are viewed without examining its degree, and the number of degrees sampled without viewing its attribute. Those may be a small percent (say 20%) and should decrease with round or in cases where substantial time is spent on either (say > 500 ms). Below I suggest an analysis that merges attribute and degree time into one measure of attribute attention.

Thank you for the insightful suggestion. On average, the attributes that are viewed (based on fixation measure) without examining their degree is 65,3%, while the number of degrees sampled without viewing their attribute is 34,7%. The overall picture is that for 45,7% of the attributes both attributes and degrees are checked, in 25,7% neither of them are checked, while in 28,5% either attribute or degree is checked, but the corresponding degree or attribute is not checked.

When plotting this measure over order, we find that, while the average sum of attributes being seen without checking the corresponding degree is decreasing over order, the opposite trend is observed for the degrees viewed in isolation.

Fig R2.2. Information in isolation. Plots of the share of attributes viewed without viewing the corresponding degree and vice-versa.

We added this additional analysis to the manuscript, which reads:

“Another level of analysis concerns whether attributes and degrees are viewed in isolation, meaning that one is viewed while the corresponding counterpart is not. In other words, we calculate the percent of attributes that are viewed without examining their degree, and the number of degrees that are viewed without examining their attribute. On average, the attributes that are viewed (based on the fixation measure) without examining their degree is 65,3%, while the number of degrees sampled without viewing their attribute is 34,7%. When studying these measures over order, we find that, while the average sum of attributes being seen without checking the respective degree is decreasing over order, the opposite trend is observed for the degrees viewed in isolation. It suggests that participants tend to focus less and less on reading the attributes over time, and more and more on noticing the level of the degrees”

2. Play down the selectivity metric from zero fixations. The problem with eye tracking is that many fixations occur randomly or to they are briefly noted and then ignored. On Table 4 the standard errors from the selectivity measures are substantially larger than the efficiency measures. One way to fix that is to define a higher cutoff for what makes a fixation, say at 500 ms. The other way is to simply focus more on what people spend more time on rather than not. Put differently, the important measure is the total time of fixations rather than the distinction between attributes with no fixations vs. some.

Thanks a lot for the comment. We agree that it is interesting to not only look at what participants do not look at, but also what they in fact look at. We have therefore decided to add measures on the number of fixations and time spent looking at the attributes. We cannot change the fixation cut-off because we do not have access to the raw data (as mentioned above).

Regarding the standard error (we assume it is Table 3 and not 4 which is referred to), we would like to highlight that the table is listing the p-values in the parentheses (the explanation of the parenthesis is also included in the title).

After following the suggestion in your comment, we have added an additional analysis and updated the manuscript such that it now reads:

“To also provide evidence on what participants in fact attend to, and not only what they do not attend to, we have performed a parallel analysis of actual fixations on attributes and degrees (each attribute and the corresponding degree is treated as one unit) for each scenario. We find that the numbers of attributes and degrees receiving fixations is significantly lower for the last ten scenarios compared to the first ten (t-test: t = 5.34; p<0.0001). Furthermore, the middle ten scenarios are also significantly different from the first ten scenarios (t = 3.45; p = 0.0003), but not different from the last ten scenarios (t = 0.83; p = 0.2022), suggesting that the selection process main takes place in the beginning. We repeated the analysis at an individual level by comparing the individual number of attributes and degrees together fixated at in the first ten scenarios with the same person’s number of fixations in the last ten scenarios. At the individual level, we confirm that the number of attributes and degrees together fixated at is significantly decreasing (paired t-test t = 3.55; p = 0.0005). Together these results underline that participants go through a process of selecting what items to attend to over the course of the study.”

3. Make a run of 21,000 observations similar to what you did in R2. The initial large estimates and small error terms in R2 lack credibility. Perhaps the measure is in milliseconds? The small errors may come from assuming that the errors within persons and tasks are uncorrelated with each other. If so, better results may come from the following analysis:

Thank you for your precision. As for the previous comment, the digits in the parentheses of Table R2 are actually the p-values, not the standard errors. We forgot to mention this in the Table note. It is now added. The measure is in milliseconds. We address the suggested analysis in the following point.

4. Predict attribute plus degree seconds with the following independent variables: fixed constants for each of the 16 attributes, fixed constants for each of the 43 respondents, continuous measures of attribute length, individual’s ranking for each attribute, and rounds. The main effects will show the relative impact of attribute length, individual ranking, and rounds. Then interactions between rankings, attribute lengths, and rounds (zero-centered) will indicate whether rankings become more predictive of decision time with rounds, whether attribute length becomes less predictive with rounds, and whether attribute rank and length and ranking interact to predict attribute time. The idea is to identify the important factors that alter decision time.

Thanks for suggesting this analysis. It is a very useful way to understand the data. Below you may find the results of the regressions. We have also included the regressions in the manuscript as Table 4, and we have added the following text:

“A final step of our analysis is to run two regressions with time spent jointly on the attribute and the corresponding degree as dependent variable. The regressions are listed in Table 4. In the first regression, model 1, we apply attribute length, stated disliking (the ranked data of attributes from most liked (=1) to least liked (=16)) and order as explanatory variables. In the second regression, model 2, we furhermore add two-way interaction effects between the explanatory variables and a three-way interaction between all of them. Both regressions control for individual dummies and the attribute dummies. All explanation variables are normalized. The first regression highlights that more time is generally spent on longer attributes (everything else kept equal), and that less time is spent on less liked attributes, while time spent is reduced over the course of the policy capturing experiment. In addition, the second regression shows that the longer time spent on more lengthy attributes is fading out over the repetition of the scenarios, suggesting that less time is spent reading the actual content of the attributes over time, but rather the time is spent observing what degrees the different attributes are scaled at.”

**Table 4. Triple interaction regression table.** Regression models of the relative impact of attribute length, individual ranking, and scenarios order on the time spent on attributes and corresponding degrees (p-values within parentheses). The three explanatory variables are normalized.

**Reviewer 2:**

My apologies for the delay in my response. It is quite a long paper and it has been a few difficult months with everyone else "going back to normal". Thank you for offering the version that shows the changes and the detailed letter with the responses to the comments. This has helped a lot. I think all my comments have been addressed in the revision. When reading the revision I thought that the introduction could have had a bit more information on the similarities and differences between the policy capturing paradigm and discrete choice experiments, but I think the current layout with this information in the discussion also works. I am glad to see that the cut-off for fixations is 100ms (which is a common threshold), instead of 200ms. I also looked through the replies to the other reviewer, and very much appreciate that you back up your responses with relevant analyses.

Thanks very much for the kind words.

The one remaining request I would still have, is to provide an overview of what each of the variables in your data-set mean (best shared along with the data-set). I had to look up the .dta format and found that it is from STATA, which I can read in R using the "haven" package, so this works out well (but you may want to add that information as well). For example, what is contained in the variables: qc1-qp1-qp2-qp3-qp4-qp5-qp6-qp7? What is in c1-c2-c3-c4-c5-c6-c7-c8-c9-c10-c11-c12-c13-c14-c15-c16-d1-d2-d3-d4-d5-d6-d7-d8-d9-d10-d11-d12-d13-d14-d15-d16-a1-a2? Some guidance on what is what would be helpful.

Thanks for your comment. We have created this detailed description of the dataset. It is printed here below, but we also attach it as a separate document to the re-submission.

**Dataset and variable description**

**Data Structure**

For each of the 44 individuals, we have PC data for 30 scenarios.

For each scenario, we have eye-tracking data for 16 criteria, 16 degrees, 2 answers.

Therefore, the dataset is composed by 44 X 30 X 16 X 16 X 2 = 46200 observations

**PC data**

id = id number randomly assigned

order = ordinal variable indicating the order on which each scenario (out of 30) was shown to each subject

group = subsample dummy

scenario = descriptive name for each of the 30 scenarios

durationinseconds = how many seconds it takes to subjects to answer to the online survey

qc1 = consent page dummy (=1 if consent was provided)

age = sociodemographic numeric variable for age

gender = dummy variable for gender

gender_long = character variable for gender

nation = dummy variable for being a local citizen or a foreigner

nationality = character variable for citizenship

edu_level = categorical variable on education level

years_work_experience = numeric variable on years of working experience following most recent education title

years_research = numeric variable on years of working experience in research

partner_choice = dummy variable indicating whether the subject is able to choose their own collaborative partner

project_choice = dummy variable indicating whether the subject is able to choose their own collaborative project

current_collab = dummy variable indicating whether the subject is currently working on a cross-sector collaborative project

item_1a - item_16a = categorical variable on the ranking position of each item provided by each subject in the ranking exercise of the PC experiment (ranging from 1 to 16)

item_1 - item_16 = categorical variable for degrees (ranging from 1 to 5) characterizing each criteria level

Answer = average numeric variable of Answer 1 and Answer 2

Answer1 = categorical variable describing the assessment likert scale (ranging from 1 to 7) for the first evaluation question of each scenario

Answer2 = categorical variable describing the assessment likert scale (ranging from 1 to 7) for the first evaluation question of each scenario

student = dummy variable

qp1 - qp7 = workplace perception questions, not used in current analysis

**Eye-tracking data**

aoiname = character variable for descriptive name of each area of interest

criteria_aoi = dummy variable indicating if area of interest is from a criterion

answers_aoi = dummy variable indicating if area of interest is from an answer

degree_aoi = dummy variable indicating if area of interest is from a degree

ttfff_ms = continuous variable indicating the time to first fixations in milliseconds for each area of interest (aoi)

timespent_fms = continuous variable indicating the time in milliseconds spent on each aoi

timespent_f = numeric variable indicating the number of times subjects look at each aoi

revisit_f_revisits = numeric variable indicating the number of times subjects revisit each aoi

fixations_count = numeric variable indicating the number of times subjects fixate each aoi

first_fixation_duration_ms = continuous variable indicating the length of time in ms of the first time subjects fixate each aoi

average_fixations_duration_ms = continuous variable indicating the avergae length of time in ms of fixations for each subjects

c1 - c16 = dummy variable indicating what of the sixteen criteria the eye-tracking data refers to

d1 - d16 = dummy variable indicating what of the sixteen degrees the eye-tracking data refers to

a1 - a2 = dummy variable indicating what of the two answers the eye-tracking data refers to

aoi_c_d = numeric variable indicating the ordering position of the areas of interest for criteria and degrees together

aoi_c = numeric variable indicating the ordering position of the areas of interest for criteria

aoi_cda = descriptive variable indicating names of areas of interest
